# Preventing neonatal sepsis in rural Uganda: a cross-over study comparing the tolerance and acceptability of three alcohol-based hand rub formulations

**DOI:** 10.1186/s12889-018-6201-3

**Published:** 2018-11-20

**Authors:** J. Ditai, M. Mudoola, M. Gladstone, J. Abeso, J. Dusabe-Richards, M. Adengo, P. Olupot-Olupot, E. D. Carrol, J. Storr, A. Medina-Lara, B. Faragher, A. D. Weeks

**Affiliations:** 1 Liverpool School of Tropical Medicine, Liverpool, UK; 2 Sanyu Africa Research Institute (SAfRI), Mbale Regional Referral Hospital, Pallisa-Kumi Road Junction, P.o Box 2190, Mbale, Uganda; 3 Sanyu Research Unit, Department of Women’s and Children’s Health, University of Liverpool c/o Liverpool Women’s Hospital, Crown Street, Liverpool, L8 7SS UK; 4 Department of Clinical Infection, Microbiology and Immunology, Institute of Infection and Global Health, University of Liverpool, 8 West Derby Street, Liverpool, L69 7BE UK; 5 WHO Consultant, Geneva, Switzerland; 6 Mbale Regional Referral Hospital Clinical Research Unit, Mbale, Uganda; 7 Health Economics Group, University of Exeter, Exeter, UK; 8 Department of Paediatrics, Mbale Regional Referral Hospital, Mbale, Uganda; 9 Tropical Clinical Trials Unit, Liverpool School of Tropical Medicine, Pembroke Place, Liverpool, L3 5QA UK; 10 Busitema University, Faculty of Health Sciences, P.o Box 1460, Mbale, Uganda; 11 S3 Global, London, UK

**Keywords:** Acceptability, Alcohol-based hand rubs, Maternal infection, Neonatal infection, Bitterant, Perfume, Hand hygiene

## Abstract

**Background:**

Neonatal sepsis causes 0.5 million deaths annually, mostly in low resource settings. Babies born in African rural homes without running water or toilet facilities are especially vulnerable. Alcohol-based hand rub (ABHR) may be used by mothers and carers as an alternative to hand washing with soap to prevent neonatal infection. However, no definite study has established the preferred formulation of hand rub for the mothers. This study aimed to assess the effects of addition of bitterants and perfume towards the acceptability of the alcohol-based hand rubs by the mothers in their homes after childbirth.

**Methods:**

This was a 3-way blinded cross-over study design. Mothers with children aged ≤3 months were recruited from immunisation clinics at 3 local health facilities in rural eastern Uganda and received 3-different ABHR formulations (in the order plain, bitterant and perfumed) packed in 100 ml bottles. Each ABHR was used for 5 consecutive days followed by a 2-day ‘washout’ period (evaluation period). Overall satisfaction with each hand rub was evaluated at the end of each week using a 7-point Likert scale.

**Results:**

A total of 43 women were recruited, whose ages ranged from 16 to 45 years (mean 26.2 years old). None of the participants normally used a hand protective lotion/cream. The three formulations were used for a mean of 5 (range 3–7) days. A significantly greater volume of the “bitterant” and “perfumed” formulations (mean 91 and 83 ml respectively) were used in comparison to the “plain” formulation (mean 64 ml). Overall satisfaction was high with all the hand rubs, but the perfumed formulation had a significantly higher overall satisfaction score [mean 6.7, range 4–7] compared with the plain **[**6.4, 3–7] and bitterant **[**6.2, 2–7] formulations.

**Conclusions:**

All the 3 ABHR formulations were well accepted with little to choose between them. The ABHR with added perfume scored highest on overall satisfaction and was used significantly more often than plain ABHR. ABHR with bitterant additive did, however, score highly and may be a preferable choice to those with concern over alcohol misuse.

**Trial registration:**

ISRCTN67852437, prospectively registered on 18/03/2018

## Background

More than 3 million neonatal deaths occur annually across the globe [1]. This accounts for two-fifths of the 7.6 million under-5-year-olds who died in 2010, with these deaths almost exclusively in low-income countries [1–3]. Neonatal sepsis results in half a million deaths each year. African community studies suggest 42% of neonatal deaths are due to infections [4].

In Uganda, with over 1.5 million live births annually, 142,000 die every year and 33% of these deaths occur in the neonatal period (0–28 days of life) [5]. Uganda ranks in the 10 highest neonatal mortality countries globally. In Uganda, the majority of newborn infections and deaths occur in the community, outside a health facility setting and are frequently unreported to the health sector [6].

Evidence shows that these infant infections are diseases of poverty, associated with poor home environments, remoteness, hunger, undernutrition, and lack of access to essential services [7–9]. Whilst some pathogens are transmitted directly, most are transmitted from toilets, animals, gardens or other unclean areas through carers’ hands [10].

Hand washing with soap at the household level is recommended as an important hand hygiene measure to prevent transmission of such infections [10, 11] but this is not so easy when there is no tap water nearby, or when the water itself is dirty or scarce. Alcohol-based hand rub (ABHR) may be used by mothers and carers as the alternative, to prevent the spread of infection to the infants. A large study comparing maternal ABHR use with normal care for prevention of newborn infective morbidity in villages around Mbale, eastern Uganda is planned. As yet, no study has established the optimal formulation of hand rub for the mothers. In European hospital settings, ABHR commonly contains bitterant to prevent its oral ingestion by alcoholics, people with alcohol-use disorders, children and mentally ill older people. This is also common in communities where alcohol ingestion is prohibited. However, this use of bitterant could affect the taste of food when hands are used for eating, as in Mbale district. An alternative would be to add perfume to the ABHR. This would increase its acceptability as well as deterring oral ingestion.

This study aimed to assess the effects of addition of bitterants and perfume on the acceptability of the alcohol-based hand rubs by the mothers in their homes following childbirth. In this study, we were interested in establishing the preferred ABHR formulation, which later could be used in a cluster randomised trial with an outcome of sepsis amongst newborns in the first 3 months after birth. In this study, women with newborns up to 3 months were included to establish inclusive experience on the tolerability and acceptability of different ABHRs for use in the future planned randomised controlled trial.

## Methods

### Setting

The study was carried out in Mbale district, Eastern Uganda. The district has 46 government-run health centres and one regional referral hospital. Mbale district has an estimated population of 568,192 people in 912 villages and a network of 2454 trained Village Health Workers as recorded from the district health office.

### The alcohol-based hand rub (ABHR)

The formulations of ABHR were manufactured for the study by Saraya East Africa Co. Ltd. based in Jinja, Uganda. At the beginning of each week, participants were given a 100 ml bottle of hand rub formulation with a label containing usage and safety instructions and the blinded formulation code (either A, B, or C). The codes were added to enable the research team to confirm the nature of the ABHR at the end of each follow-up period. The bottle contents were stated as being an ABHR, but it was not stated which additives were used. The following formulations were compared: “A” was plain ABHR containing ethanol 80% (Alsoft V, Saraya East Africa Ltd); “B” was Alsoft V with added bittering agent, and “C” was Alsoft V with an added floral perfume.

### Recruitment

All women with children aged utmost 3 months attending infant immunisation clinics in three lower health facilities in Mbale district, eastern Uganda during the week commencing 2nd June 2015 were screened for eligibility. All women were included except those who were currently using antiseptic hand wash at home and wished to continue its use.

### Design

This was a 3-way blinded non-randomised cross-over study design. Each participant received the ABHRs in the order “A” (plain), “B” (bitterant), “C” (perfumed of floral scent). All three formulations were provided in identical 100 ml bottles and colour with instructions to use for 5 consecutive days, followed by a 2-day ‘washout’ period in which any or no hand rub could be used. Participants were asked to return to the health centre with their current ABHR bottle at the end of each week; the research assistant then completed the study evaluation from the participant’s perspective (participant’s reported practice) and provided observer evaluation of the participant in line with the WHO protocol [12]. Hand rub bottles from the previous week were retrieved and participants received the next ABHR formulation at each evaluation visit. The frequency of ABHR use was assessed, the amount of the formulation remaining in the bottle measured and any remaining hand rub was given back to women only after the third formulation-follow up had been completed. The volume of ABHR consumed was physically calculated by subtracting the remaining volume of ABHR in the bottle at the time of evaluation from the total volume in the bottle at the start (100mls). Those who failed to attend for follow-up were contacted by mobile phone or through the village health worker and the visit was rescheduled to another appropriate time and place, including the option of a home visit if preferred.

**Fig. 1:**
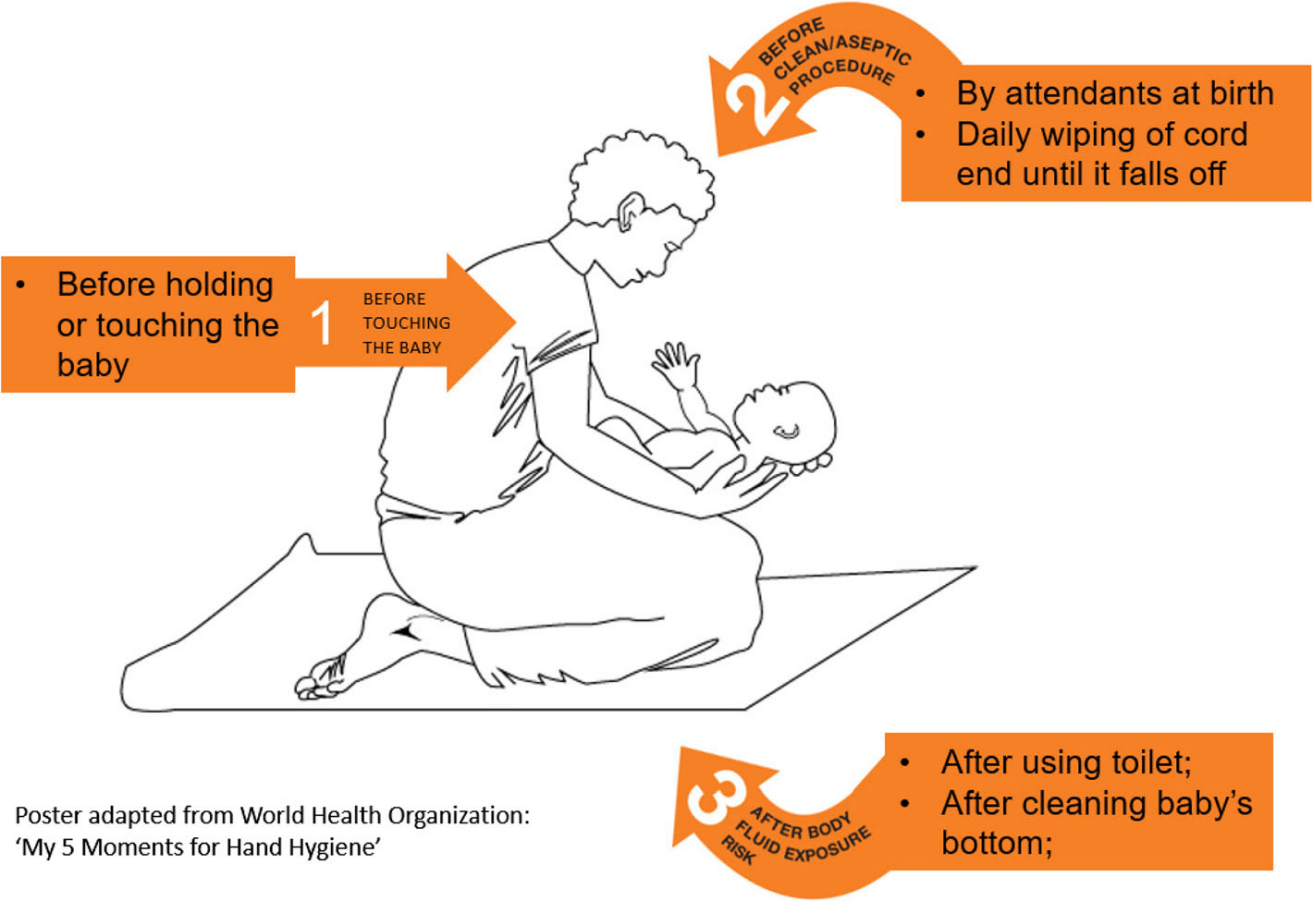
The ‘3 Moments for community neonatal hand hygiene’ poster developed for BabyGel study. This shows an illustrative and diagrammatic representation of the key moments of hand hygiene for newborns in the community

Participants were instructed by the researchers at the time of recruitment on how to use the hand rubs based on the adopted ‘3 moments of hand hygiene for community neonatal care’ (Fig. 1) derived from the WHO ‘5 moments for hand hygiene’ in non-hospital settings [10]. The three moments included; 1- before touching the baby (*eg before breastfeeding*), 2- before a clean/aseptic procedure (*eg cleaning of the umbilical cord end until it falls off*), 3- after body fluid exposure *(eg after using the toilet, after cleaning baby’s bottom, after changing the baby’s diapers/nappy). The moments’ poster was translated into the local language (Lumasaba).* Pictorial instructions on how to hand rub were provided to each participant.

### Data collection

**Fig. 2:**
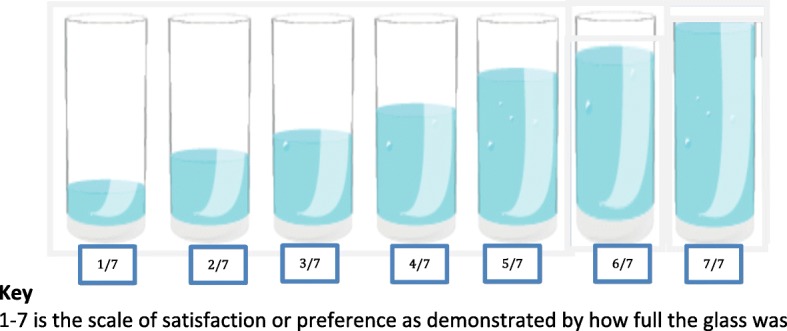
The Glass model for Likert scale evaluation of the opinion of Carers/ mothers after use of the test product. The glass models were labelled from 1 to 7 consistent with the filling levels and each participant was asked to indicate where she felt it is the best option for her following use of the product

Data were collected using the WHO validated tool designed to evaluate the acceptability and tolerability of different ABHRs [12]. The primary outcome measure was the participant’s overall evaluation of the three ABHRs on a 7-point Likert scale, demonstrated to participants using water filling levels in a glass model for each point scale, as shown in Fig. 2. At each evaluation visit, a participant was asked to provide an overall evaluation following the ABHR use according to the WHO protocol [12]. Data were also collected on some participant demographics, the frequency of ABHR use, ABHR volume consumed, the opinion of the hand rubs (colour, smell, texture, ease of use, drying), skin condition after use, and factors the participants both liked and disliked about the ABHR. The frequency of ABHR Use was assessed by asking women the number of times the hand rub was used at the last 10 times that they did particular activities related to the baby, like breastfeeding, changing diapers, after using toilets etc.

The data were collected on paper case report forms and then transferred onto a secure, password-protected database for statistical analysis.

### Sample size

A sample size of 40 mothers or carers with children utmost 3 months old and attending the immunisation clinics was used. WHO protocol recommends approximately 40 volunteer participants using at least 30 ml of product per day to participate in acceptability and tolerability studies for ABHR use [12] and previous studies have adopted similar sample size [13].

### Statistical analysis

Participant characteristics and product performance measures were summarised using means and standard deviations (± range) for continuous variables and frequency counts (with percentages) for categorical measures. User comparisons of the performances of the three hand rub formulations were summarised using mean differences with their 95% confidence intervals. Observer evaluations of the impact of the formulations on participants’ skin condition were summarised using frequency counts and percentages; as only one participant reported a (very minor) problem, no formal statistical comparisons were performed for these variables. Time spent away from home compound by participants was measured using an ordered categorical scale and summarised using frequency counts with their percentages. Differences between the formulations on this measure were evaluated using the Wilcoxon matched-pairs rank sum test but mean scores were also computed to help inform the interpretation of these differences. Preferences for the two bottle sizes (1-Litre large and 100 ml small) used were summarised using frequency counts with their percentages; differences between these preferences were evaluated using the McNemar test. All analyses were conducted using the SPSS 22.0 (IBM Corp, Chicago, USA). Statistical significance was set at the conventional 5% level.

## Results

### Participants’ characteristics

**Fig. 3:**
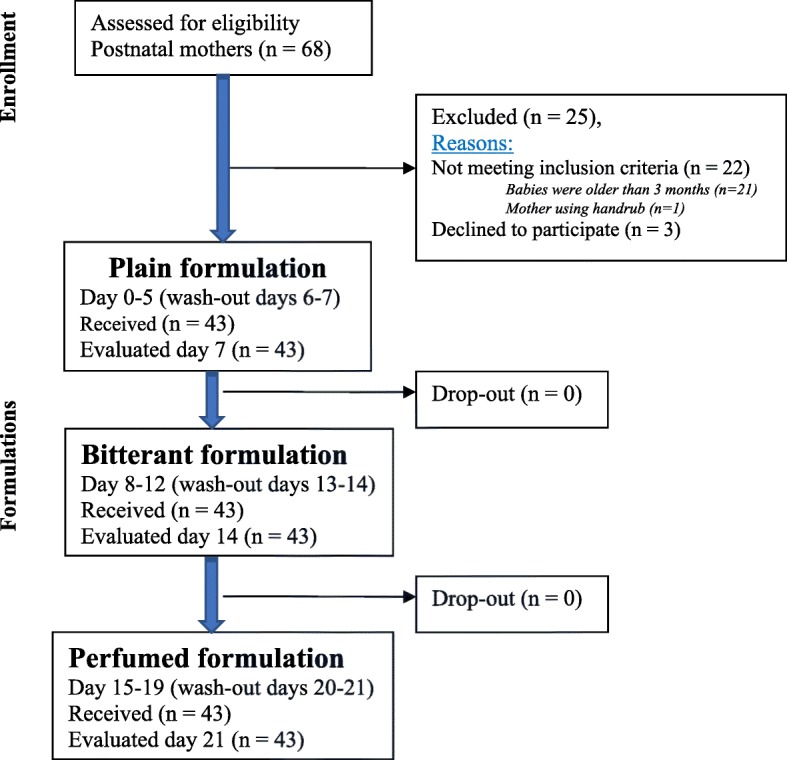
Participants flow in the study. This shows the potential participants screened and those recruited and the allocation to the ABHR formulations

Table 1 shows that a total of 43 women with children aged utmost 3 months were recruited into this study from the immunisation clinics at three lower health centres (HC) (One HC4 facility at the county level and two HC3 facilities at sub-county level) in rural eastern Uganda. Figure 3 shows the flow of participants in the study.

**Table 1:** Participant characteristics

Characteristic			
Sample size			43
Date of enrolment		range	2/6/15: 5/6/15
Age (years)		mean (s.d.) [range]	26.2 (7.1) [16: 45]
Skin	brown	*n* (%)	7 (16.3)
	dark brown	*n* (%)	22 (51.2)
	black	*n* (%)	14 (32.6)
Present season	humid	*n* (%)	43 (100)
Do you normally use a protective hand lotion /cream (outside test period)?	never	*n* (%)	43 (100)
Do you develop irritative dermatitis?	never	*n* (%)	35 (81.4)
	sometimes	*n* (%)	7 (16.3)
	always	*n* (%)	1 (2.3)
Do you develop atopic dermatitis?	yes	*n* (%)	2 (4.7)
	no	*n* (%)	41 (95.3)
Do you develop rhinitis / allergic conjunctivitis?	yes	*n* (%)	3 (7.0)
	no	*n* (%)	40 (93.0)
Are you asthmatic?	yes	*n* (%)	1 (2.3)
	no	*n* (%)	42 (97.7)
Do you have a known intolerance to alcohol?	no	*n* (%)	43 (100)
Do you think you can improve your own hand hygiene?	yes	*n* (%)	42 (97.7)
	perhaps	*n* (%)	1 (2.3)
Did any of the following problems make it difficult to use the hand rub (1 = always; 7 = never)?			
Forgetfulness	1 (always)	*n* (%)	0
	2	*n* (%)	3 (7.0)
	3	*n* (%)	4 (9.3)
	4	*n* (%)	3 (7.0)
	5	*n* (%)	1 (2.3)
	6	*n* (%)	3 (7.0)
	7 (never)	*n* (%)	29 (67.4)
		*mean (s.d.)*	*6.0 (1.7)*
Lack of time	1 (always)	*n* (%)	0
	2	*n* (%)	2 (4.7)
	3	*n* (%)	0
	4	*n* (%)	1 (2.3)
	5	*n* (%)	1 (2.3)
	6	*n* (%)	4 (9.3)
	7 (never)	*n* (%)	35 (81.4)
		*mean (s.d.)*	*6.6 (1.2)*
Damaged skin	7 (never)	*n* (%)	43 (100)
		*mean (s.d.)*	*7.0 (0)*

Every participant received each of the formulations over three consecutive 7-day periods (3 weeks), in the order ‘plain’, ‘bitterant’, ‘perfumed’. The study was conducted between 2nd and 16th June 2015, which was categorised as the “humid” season. The mean age of the participants was 26.2 years (range 16 to 45 years). Just over half of the women (22; 51.2%) were classified as having “dark brown” skin, almost one third (14; 32.6%) as having “black” skin, and the remainder (7; 16.3%) as having “brown” skin. None of the participants normally used a hand lotion/cream. A small number of women indicated experiencing conditions compatible with irritative or atopic dermatitis, rhinitis, or allergic conjunctivitis and or asthma. No woman reported an intolerance to alcohol. Just one woman considered that the ABHR could improve her own hand hygiene.

### Evaluation of formulations by participants

Table 2 shows that all the three formulations were used for an average of 5 (range 3–7) of the 7 evaluation days. There was a gradual increase in the volume used over time, with more amount for the perfumed and bitterant formulations (mean 83 ml and 91 ml respectively) than for the plain formulation (mean 64 ml). All women reported that the three formulations had changed their hand hygiene practices. The bitterant and perfumed formulations were used more frequently than the plain formulation for all activities assessed, with the single exception of changing the baby’s nappy/diaper. However, no statistically significant differences were observed between the bitterant and perfumed formulations for any of the activities. Overall, all the ABHRs formulations were well accepted by women but the ABHR with perfume additive was favoured, mean score 6.7 (range 4–7) more than the plain ABHR, mean 6.4 (range 3–7) and ABHR with the bitterant additive, mean 6.2 (range 2–7). The preference was assessed based on colour, smell, texture, irritation, drying effect, ease of use, the speed of drying and application of the ABHR formulation for hand hygiene (Table 2).

**Table 2:** Comparison of products: (1) User observations

Characteristic	Product; mean (s.d.) [range]			Differences (95% CIs)				
	A (Plain)	B (Perfumed)	C (Bitterant)	A vs. B	A vs. C	B vs. C		
Time since enrolment (days)	7 (0) [7: 7]	14 (0) [14: 14]	21 (0) [21: 21]	0	0	0		
Total amount of product used (ml)	64 (27) [5: 100]	83 (17) [25: 100]	91 (11) [50:100]	*19 (10*: 28)^*^	*27 (18*: 35)^*^	*8 (2*: 14)^*^		
For how many days did you use product?	5.1 (0.5) [3: 7]	5.0 (0.3) [4: 6]	5.1 (0.4) [4: 7]	− 0.1 (− 0.2: 0.1)	0 (− 0.2: 0.2)	− 0.1 (− 0.1: 0.2)		
Study *has* changed hand practice.	42 (100)^**^	43 (100)	43 (100)	0	0	0		
Number of times hand rub was used at last 10 times:								
when breastfeeding your baby	7.1 (2.4) [3: 10]	7.6 (2.2) [3: 10]	7.8 (1.9) [4: 10]	0.5 (− 0.3: 1.3)	*0.7 (0.0*: 1.4)^*^	0.2 (− 0.5: 0.9)		
when changing your baby’s diaper	6.7 (2.7) [1: 10]	7.0 (2.4) [1: 10]	6.7 (2.5) [2: 10]	0.3 (− 0.7: 1.3)	−0.1 (− 0.9: 0.8)	−0.4 (− 1.2: 0.4)		
before friend/family handled your baby	4.7 (2.9) [0: 10]	5.1 (2.7) [0: 10]	5.8 (2.8) [0: 10]	0.4 (− 0.5: 1.3)	*1.1 (0.1*: 2.1)^*^	0.7 (− 0.4: 1.7)		
before you handled your baby	6.1 (2.9) [1: 10]	7.5 (2.5) [2: 10]	7.3 (2.3) [2: 10]	*1.5 (0.3*: 2.6)^*^	*1.2 (0.2*: 2.2)^*^	− 0.3 (− 1.1: 0.5)		
After you went for a “short call” or to urinate	5.3 (2.8) [1: 10]	5.5 (2.6) [2: 10]	6.1 (2.7) [2: 10]	0.3 (− 0.5: 1.1)	0.9 (− 0.1: 1.8)	0.6 (− 0.4: 1.6)		
After you went for a “long call” or to defeacate	4.5 (2.6) [1: 10]	5.6 (2.9) [1: 10]	5.3 (2.7) [1: 10]	*1.1 (0.2*: 2.0)^*^	0.8 (− 0.1: 1.7)	− 0.3 (− 1.4: 0.8)		
Opinion of test product for hand hygiene:								
colour (1 = unpleasant, 7 = pleasant)	6.4 (1.0) [2: 7]	6.4 (0.9) [2: 7]	6.5 (0.8) [4: 7]	0.1 (− 0.2: 0.3)	0.1 (− 0.2: 0.4)	0.0 (− 0.3: 0.3)		
smell (1 = unpleasant, 7 = pleasant)	5.6 (1.6) [1: 7]	5.9 (1.3) [2: 7]	5.1 (2.0) [1: 7]	0.3 (− 0.3: 0.9)	− 0.6 (− 1.3: 0.2)	*− 0.9 (− 1.5*: − 0.2)^*^		
texture (1 = very sticky; 7 = not sticky at all)	6.1 (1.2) [1: 7]	6.3 (1.0) [2: 7]	6.0 (1.4) [1: 7]	0.2 (− 0.2: 0.6)	−0.1 (− 0.6: 0.5)	−0.3 (− 0.8: 0.3)		
irritation (1 = very irritating; 7 = not irritating)	6.0 (1.3) [2: 7]	6.1 (1.3) [3: 7]	6.2 (1.3) [1: 7]	0.1 (− 0.5: 0.6)	0.2 (− 0.3: 0.8)	0.2 (− 0.4: 0.7)		
drying effect (1 = very much; 7 = not at all)	2.8 (2.0) [1: 7]	3.3 (2.3) [1: 7]	2.9 (2.4) [1: 7]	0.5 (− 0.4: 1.4)	0.1 (− 0.7: 1.0)	−0.3 (− 1.3: 0.6)		
ease of use (1 = very difficult; 7 = very easy)	6.5 (1.0) [3: 7]	6.7 (0.6) [5: 7]	6.6 (1.0) [1: 7]	0.2 (− 0.1: 0.5)	0.1 (− 0.3: 0.5)	−0.1 (− 0.4: 0.2)		
speed of drying (1 = very slow; 7 = very fast)	6.5 (0.6) [5: 7]	6.5 (0.8) [3: 7]	6.7 (0.7) [3: 7]	0.0 (− 0.2: 0.2)	0.2 (0.0: 0.5)	0.2 (− 0.1: 0.5)		
application (1 = very unpleasant; 7 = very pleasant)	6.4 (0.9) [4: 7]	6.6 (0.6) [5: 7]	6.7 (0.5) [5: 7]	0.3 (0.0: 0.6)	*0.3 (0.0*: 0.6)^*^	0.1 (− 0.2: 0.3)		
overall evaluation (1-dissatisfied; 7 = very satisfied)	6.4 (0.9) [3: 7]	6.7 (0.7) [4: 7]	6.2 (1.1) [2: 7]	*0.3 (0.0*: 0.5)^*^	− 0.2 (− 0.5: 0.1)	*−0.5 (− 0.8*: − 0.2)^*^		
Do you think hand rub makes it easier to keep your hands clean? (1 = yes, absolutely; 7 = not at all)	1.5 (1.1) [1: 6]	1.3 (0.5) [1: 2]	1.2 (0.5) [1: 3]	− 0.2 (− 0.5: 0.1)	*−0.3 (− 0.6*: 0.0)^*^	*−* 0.1 (− 0.3: 0.0)		
Self-assessment of skin on hands								
appearance (1 = abnormal; 7 = normal)	7.0 (0.2) [6: 7]	7.0 (0.2) [6: 7]	7.0 (0.0) [7: 7]	0.0 (− 0.1: 0.1)	0.0 (0.0: 0.1)	0.0 (0.0: 0.1)		
intactness (1 = abnormal; 7 = normal)	7.0 (0.2) [6: 7]	7.0 (0.0) [7: 7]	7.0 (0.0) [7: 7]	0.0 (0.0: 0.1)	0.0 (0.0: 0.1)	–		
moisture content (1 = abnormal; 7 = normal)	6.9 (0.4) [5: 7]	7.0 (0.0) [7: 7]	7.0 (0.0) [7: 7]	0.1 (0.0: 0.2)	0.1 (0.0: 0.2)	–		
sensation (1 = abnormal; 7 = normal)	7.0 (0.0) [7: 7]	6.9 (0.3) [5: 7]	7.0 (0.0) [7: 7]	0.0 (− 0.1: 0.0)	–	0.0 (0.0: 0.1)		
Frequency of ABHR Use and ABHR Bottle preference								
In first 3 months after childbirth, how often on average do you leave your home compound?	never	*n* (%)	1 (2.3)	3 (7.0)	0			
	1–5 times	*n* (%)	23 (53.5)	20 (46.5)	17 (39.5)			
	6–10 times	*n* (%)	11 (25.6)	9 (20.9)	15 (34.9)	*p* = 0.562	*p = 0.051* ^*+*^	*p* = 0.320
	most weeks	*n* (%)	5 (11.6)	7 (16.3)	8 (18.6)			
	most days	*n* (%)	3 (7.0)	4 (9.3)	3 (7.0)			
	mean score		2.67	2.74	2.93	–	–	–
Which size of bottle do you prefer?	large	*n* (%)	33 (76.7)	34 (79.1)	33 (76.7)	*p* = 1.000	*p* = 1.000	*p* = 1.000
	small	*n* (%)	10 (23.3)	9 (20.9)	10 (23.3)			

**: not recorded for 1 participant

*: *p* < 0.05

‡: Average times compound left – Wilcoxon matched-pairs rank sum test; Which size of bottle do you prefer – McNemar test

The women reported that they left their home compound when using the different formulations, but the differences between the three formulations on this item were small and probably not of any clinical relevance. This finding might help the mother in planning for the amount of ABHR needed outside the home, applying the ABHR to opportunities for hand hygiene outside homes which would ultimately improve adherence and acceptability beyond homes. Three-quarters of the women reported a preference for the large (1 l) bottle rather than the small (100 ml) bottle for all three formulations.

### Observer evaluation of the test products

Table 3 shows that skin condition was rated as normal for all women for all categories (redness, scaliness, fissures and skin scale) both before and after the use of all three formulations.

**Table 3:** Comparison of products: (2) Observer evaluation

Characteristic		A (Plain)		B (Perfumed)		C (Bitterant)		Differences (95% CIs)^‡^		
		Before	After	Before	After	Before	After	A vs. B	A vs. C	B vs. C
Observer evaluation of skin condition:										
Redness no redness	*n* (%)	42 (97.7)	43 (100)	43 (100)	43 (100)	43 (100)	43 (100)	–	–	–
slight redness or blotchiness	*n* (%)	1 (2.3)	0	0	0	0	0			
Scaliness no scaliness	*n* (%)	43 (100)	43 (100)	43 (100)	43 (100)	43 (100)	43 (100)	–	–	–
Fissures no fissure	*n* (%)	43 (100)	43 (100)	43 (100)	43 (100)	43 (100)	43 (100)	–	–	–
Skin scale no observable scale or irritation of any kind	*n* (%)	42 (97.7)	43 (100)	43 (100)	43 (100)	43 (100)	43 (100)	–	–	–
occasional scale not necessarily uniformly distributed	*n* (%)	1 (2.3)	0	0	0	0	0			

## Discussion

In general, the differences between the ratings of the three formulations were small, but the bitterant and perfumed formulations were preferred over the plain formulation. The perfumed formulation scored significantly higher than both the plain and bitterant varieties in the overall evaluation. It also emerged as slightly better than the bitterant formulation in terms of smell, with women favouring the smell of the perfumed formulation more than that of the bitterant and plain formulations. The progressive increase in the volume of ABHR use over time could be explained either by the preference of a particular type of ABHR formulation or habit formation over time which could have increased familiarity with the product. Hence, we proposed the ABHR to be introduced in the late weeks of pregnancy for mothers’ habit formation and familiarity in the planned trial and future use. In this study, there was no reported change in skin colour after using the ABHR. The skin colour change might be important in mainly brown or light-skinned population to guide the participants and observers in the occurrence of inflammatory reaction associated with the ABHR [13]. However, it didn’t prove useful in this African population.

To our knowledge, this is the first study to directly compare preferences of the different formulations in a blinded fashion in this setting. The results suggest that the perfumed ABHR is favoured in these communities. In clinical settings, formulations containing bitterant are often preferred due to safety concerns about intentional ingestion of the ABHR by people with alcohol misuse disorder [14, 15]. The WHO recommends that in cases where the risk of ingestion is very high (e.g. in paediatric or confused patient situations), bitterants could be added to make ABHRs unpalatable. At the outset, the study team had concerns that the regular use of an ABHR containing a bitterant could adversely affect the taste of food in populations where food is consumed with the hands. We found no evidence of this however from the extra notes and it is likely that the ritualistic pre-meal handwashing that is very socially important in Uganda prevents any bitterant transferring to the food.

Discussions on likely unacceptability of ABHRs on grounds of religion are widely debated [16] and it has been argued that Muslim health workers might find them difficult to use as contact with alcohol is religiously forbidden [17, 18]. The consensus, however, is that there is no skin absorption to cause concern on these grounds [19]. In the setting where we conducted this study, 15% of the population are Muslim. None of our participants expressed any concerns about the hand rub in relation to their religion from the extra notes taken.

Acceptability was high and the participants expressed their willingness to use the hand rubs at the time of the research and in the future. In 2015, a study exploring the same question amongst Swiss healthcare workers also reported high acceptance rates [13].

The study is limited by the non-randomized order of ABHR formulation use as described earlier. The non-random nature doesn’t give an explanation for the increasing volumes of ABHR use as a result of habit formation. Further, alongside the observer evaluation, which was objective, we had the self-reported evaluation of the tolerability and acceptability of ABHR use from participants and might have led to respondent’s bias. For example, the volume of the ABHR use was entirely reliant on the honesty of the participants. We assumed that participants used the ABHR correctly instead of pouring it or using it for other purposes.

## Conclusion

We, therefore, report that ABHRs were well accepted and could be used in interventions to improve hand hygiene in rural home settings in Uganda, with a preference for a perfumed or bitterant additive. In view of the potential for misuse, the formulation with bitterant was selected for community use in the planned randomised controlled trial. We recommend the early introduction of ABHR to mothers before delivery for habit formation and familiarity with the ABHR.

## Abbreviations

ABHR: Alcohol-Based Hand Rub
SAE: Severe Adverse Event
UNCST: Uganda National Council for Science and Technology
VHW: Village Health Worker
WHO: World Health Organisation
